# Quantitative assessment of the association between APC promoter methylation and breast cancer

**DOI:** 10.18632/oncotarget.9354

**Published:** 2016-05-13

**Authors:** Keli He, Li Zhang, Xinghua Long

**Affiliations:** ^1^ Zhongnan Hospital of Wuhan University, Wuhan, 430071, China; ^2^ Department of Clinical Laboratory, The First People's Hospital of Changde City, Changde, 415003, China

**Keywords:** adenomatous polyposis coli, APC, methylation, meta-analysis, breast cancer

## Abstract

Adenomatous polyposis coli (APC) is an important tumor suppressor gene in breast cancer. However, there were inconsistent conclusions in the association between APC promoter methylation and breast cancer. Hence, we conducted a meta-analysis to quantitatively assess the clinicopathological significance and diagnosis role of APC methylation in breast cancer. In total, 3172 samples from 29 studies were performed in this study. The odds ratio (OR) of APC methylation was 5.92 (95% CI = 3.16–11.07) in breast cancer cases compared to controls,. The APC promoter methylation was associated with cancer stage (OR = 0.47, 95% CI = 0.28–0.80, *P* = 0.006), lymph node metastases (OR = 0.55, 95% CI = 0.36–0.84, *P* = 0.005) and ER status (OR = 1.34, 95% CI = 1.03–1.73, *P* = 0.003) in breast cancer. Furthermore, the sensitivity and specificity for all included studies were 0.444 (95% CI: 0.321–0.575, *P* < 0.0001) and 0.976 (95% CI: 0.916–0.993, *P* < 0.0001), respectively. These results suggested that APC promoter methylation was associated with breast cancer risk, and it could be a valuable biomarker for diagnosis, treatment and prognosis of breast cancer.

## INTRODUCTION

Breast cancer is the first leading cause of cancer-related death among women [1, 2]. In worldwide, approximately 1.3 million women were diagnosed with breast cancer each year [2]. As we know that breast cancer onset and progression are caused by a series of epigenetic and genetic changes. DNA methylation is a commonly observed epigenetic modification in human malignancies [3, 4]. Methylation in tumor suppressor gene is one of the most common methylation, in cancer, including breast cancer [5, 6]. Therefore, it is important to identify the role of suppressor genes methylation in breast cancer and gain a better understanding of the underlying pathogenesis of breast cancer.

The adenomatous polyposis coli (APC) gene located at chromosomal band 5q21–q22 is a classical tumor suppressor gene [7]. APC inactivation leads to dysfunction of β-catenin protein degradation, and then activates Tcf/Lef and causes abnormal transcription of oncogenens, such as c-myc, c-jun and cyclin D1, finally leads to carcinogenesis [8]. Methylation in APC gene has been investigated in several types of malignancies, including colorectal cancer [9], prostate cancer [10], hepatocellular carcinoma [11], and breast cancer [12]. Even though there were lots of investigations, the relationships between APC promoter methylation and breast cancer are still controversial. Z Jin [12] and Masaru Shinozaki [13] thought APC methylation was related to breast cancer (*p* < 0.05), but So Yeon Park [14] and Susan R. Sturgeon [15] thought APC methylation had no relationship with breast cancer (*P* > 0.05). Therefore, we preformed a meta-analysis to quantitatively assess the association of APC promoter methylation with breast cancer risk and the clinical characteristics observed in breast cancer patients. Additionally, we comprehensively evaluated the diagnostic value of APC methylation for breast tumors, in order to provide evidence for the future application of APC in the prevention, diagnosis and treatment of breast cancer.

## RESULTS

### Identification of relevant studies

**Figure 1:**
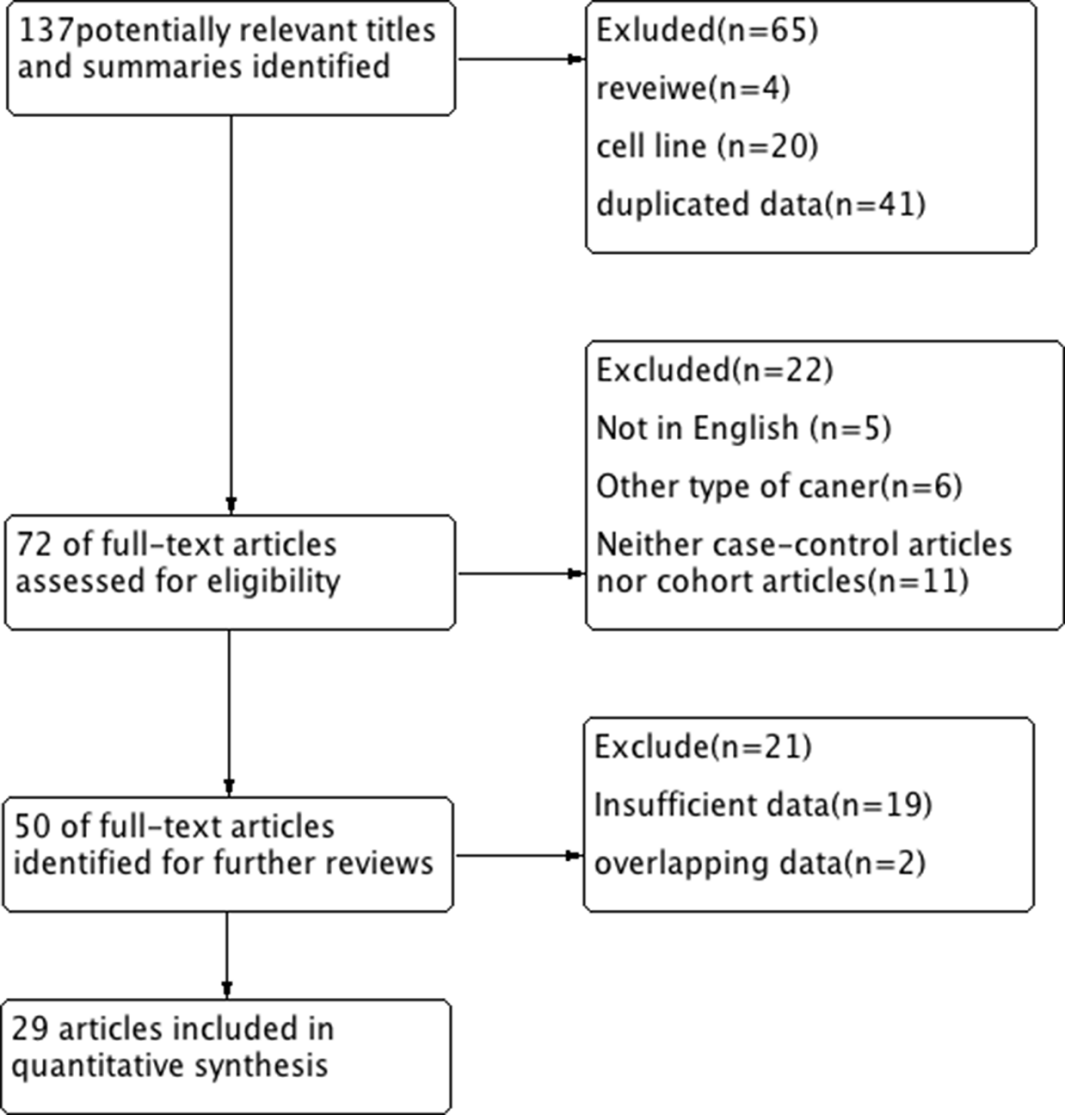
Flow diagram of the study selection process

A total of 29 eligible studies were included in the pooled analyses based on the search method as described above [12, 14–42]. As the studies by Auwera (2009 and 2010) and Hoque (2006 and 2009) investigated the patients from different cities, we did not exclude them. The flow chart in Figure 1 summarized the study selection process.

### Study characteristics

A total of 3172 samples from 29 articles were performed in this meta-analysis. Among 29 studies, there were 26 case-control studies [12, 14, 15–33, 35, 37–40] and 3 cohort studies [35, 36, 41]. Interestingly, 12 studies [12, 14, 15, 18, 19, 22, 24, 26–28, 32, 38] among 26 case-control studies also did the cohort analyses. Therefore, there were 26 case-control studies for the relationship of APC promoter methylation and breast cancer risk, and 15 cohort studies related with the association between APC methylation and clinicopathological characteristics of breast cancer. The major characteristics of the studies included in this meta-analysis were shown in Table 1.

**Table 1:** Characteristics of studies included in the meta-analysis

Study	Year	Country	Ethnicity	Case		Control		Control type	Method
				M	UM	M	UM		
Auwera et al. 2	2009	Belgium	Caucasian	23	56	1	18	Blood	QMSP
Auwera et al.	2010	Belgium	Caucasian	31	25	20	36	Tissue	QMSP
Brooks	2010	USA	Mix	1	49	6	142	Blood	QMSP
Cho et al.	2010	USA	Caucasian	21	19	12	15	Tissue	QMSP
Dulaimi et al.	2004	USA	Caucasian	15	19	0	12	Tissue	MSP
Hoque et al.	2006	Senegal	Africa	14	79	0	38	Blood	QMSP
Hoque et al.	2009	Italy	Caucasian	56	56	0	32	Tissue	QMSP
Jeronino et al.	2008	Portugal	Caucasian	55	11	30	13	Tissue	QMSP
Jin et al.	2001	Japan	Asia	18	32	0	21	Tissue	MSP
Jing et al.	2010	China	Asia	14	36	0	50	Blood	MSP
Jung et al.	2013	Korea	Asia	19	41	0	60	Tissue	QMSP
Lee et al.	2004	Korea	Asia	14	18	0	19	Tissue	MSP
Lewis et al.	2005	USA	Caucasian	15	12	23	59	Tissue	MSP
Martins et al.	2011	Portugal	Caucasian	144	34	18	15	Tissue	QMSP
Matusckek et al.	2010	Germany	Caucasian	25	60	2	22	Blood	QMSP
Müller et al.	2003	Austria	Caucasian	6	20	0	10	Blood	QMSP
Pang et al.	2014	Australian	Caucasian	39	41	0	18	Tissue	QMSP
Park et al.	2011	Korea	Asia	31	54	2	28	Tissue	QMSP
Parrela et al.	2004	Italy	Caucasian	11	43	1	9	Tissue	MSP
Prasad et al.	2008	India	Caucasian	6	26	0	5	Tissue	MSP
Rykova et al.	2006	Russian	Caucasian	4	6	0	6	Blood	MSP
Shinozak et al.	2005	USA	Caucasian	74	77	0	10	Tissue	MSP
Sturgeon et al.	2012	USA	Caucasian	9	236	10	186	Blood	QMSP
Sunami et al.	2008	USA	Caucasian	51	14	-	-	-	MSP
Swellam et al.	2015	Egypt	Africa	113	8	6	70	Blood	MSP
Virmani et al.	2001	USA	Caucasian	34	43	3	28	Tissue	MSP
Wojdacz et al.	2011	Denmark	Caucasian	23	157	13	95	Blood	QMSP
Xu et al.	2010	USA	Mix	412	439	-	-	-	QMSP
Tserga et al.	2012	Greece	Caucasian	22	27	-	-	-	QMSP

M, methylation; UM unmethylation; MSP, Methylation-Specific PCR; QMSP, Quantitative real time Methylation Specific.

### Association between APC promoter methylation and breast cancer risk

**Figure 2:**
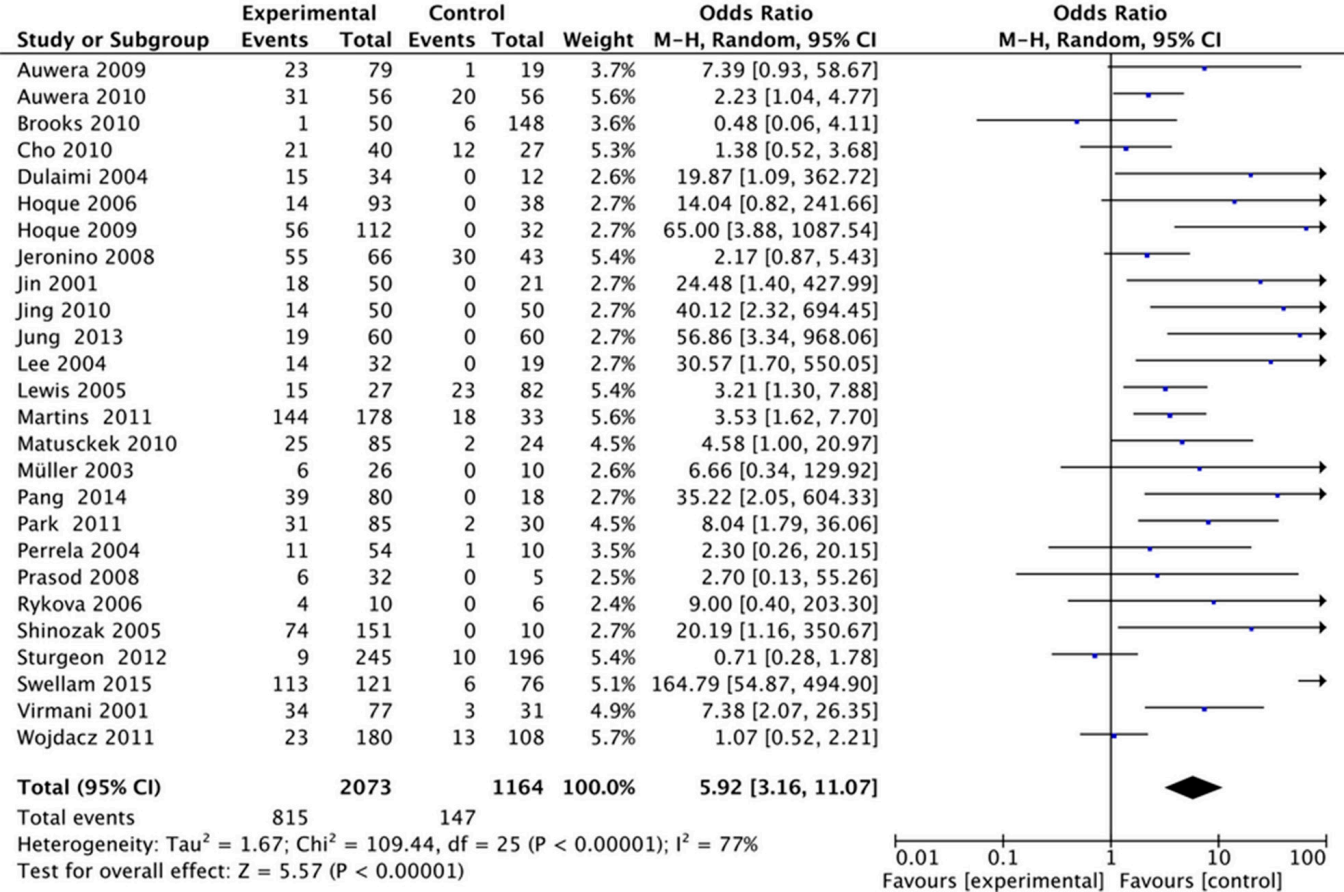
Forest plots of association between APC promoter methylation and breast cancer

In this study, we found that the frequency of APC methylation was significantly higher in breast cancer than normal controls. The pooled OR from 26 studies including 2073 breast cancers and 1164 controls was 5.92 with 95% CI: 3.16–11.07 (Figure 2). With significant heterogeneity across the included studies (*I*^2^ = 77%), the stratification analyses of this meta-analysis were performed based on methods for detecting methylation, control types and ethnicities. In the stratified analysis by method, significantly increased breast cancer risk was associated with APC methylation by both QMSP method (OR = 3.11, 95% CI = 1.72–5.62, *P* = 0.0002) and MSP method (OR = 13.38, 95% CI = 4.34–41.25, *P* < 0.00001). Stratified analysis by control type showed that significantly increased risk was associated with APC methylation in tissue samples (OR = 4.56, 95% CI = 2.63–7.90, *P* < 0.00001), and blood samples (OR = 7.42, 95% CI = 1.55–35.48, *P* = 0.01). For stratified analysis by ethnicity, the APC methylation showed statistically significant association with increased risks of breast cancers in Caucasians (OR = 3.08, 95% CI = 1.92–4.96, *P* < 0.00001) and in non-Caucasians (OR = 18.75, 95% CI = 4.12–85.28, *P* < 0.00001). Details are shown in Table 2.

**Table 2:** Subgroup analysis APC promoter methylation in breast cancer

	Case		Control		OR [95% CI]	*P*	*I*^2^ (%)	Model
	M	UM	M	UM				
Total	815	1258	147	1017	5.92 [3.16, 11.07]	< 0.00001	77	Random
Method								
MSP	318	320	33	316	13.38 [4.34, 41.25]	< 0.00001	72	Random
QMSP	497	938	114	728	3.11 [1.72, 5.62]	0.0002	65	Random
Ethnicity								
Caucasian	591	941	133	589	3.08 [1.92, 4.96]	< 0.00001	52	Random
non-Caucasian	224	317	14	428	18.75 [4.12, 85.28]	< 0.0001	75	Random
Control type								
Blood	231	667	32	495	7.42 [1.55, 35.48]	0.01	89	Random
Tissue	584	600	115	522	4.56 [2.63, 7.90]	< 0.00001	52	Random

M, methylation; UM unmethylation; MSP, Methylation-Specific PCR; QMSP, Quantitative real time Methylation Specific.

### Association between APC prompter methylation and breast cancer clinicopathological characteristics

**Figure 3:**
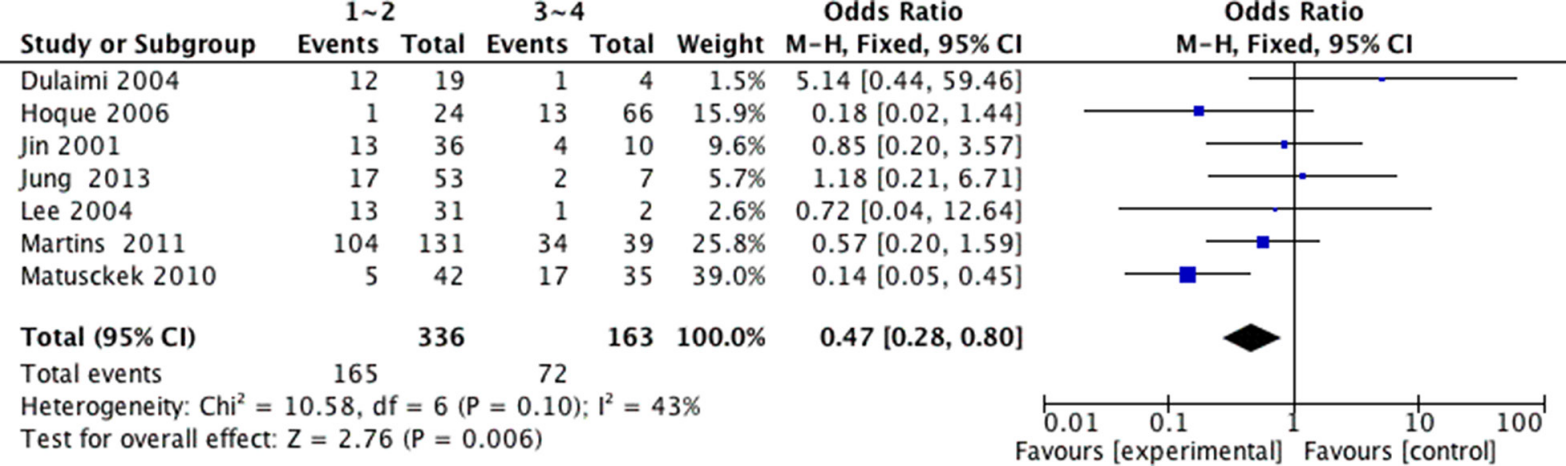
Forest plots of association between APC promoter methylation and cancer stage in breast cancer

**Figure 4:**
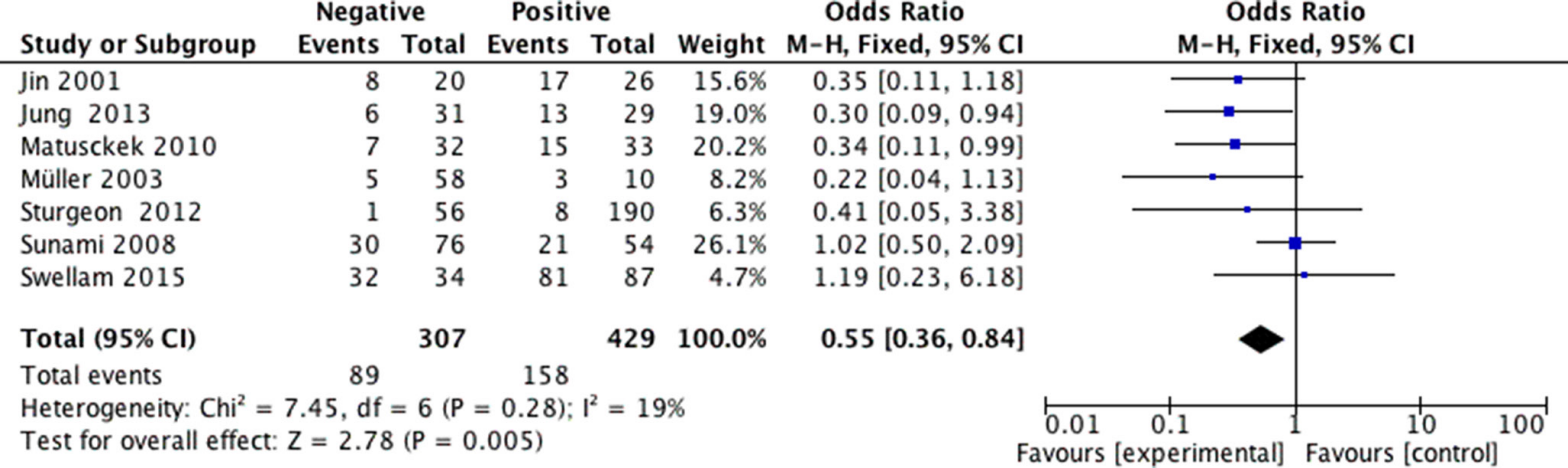
Forest plots of association between APC promoter methylation and lymph node metastasis in breast cancer

**Figure 5:**
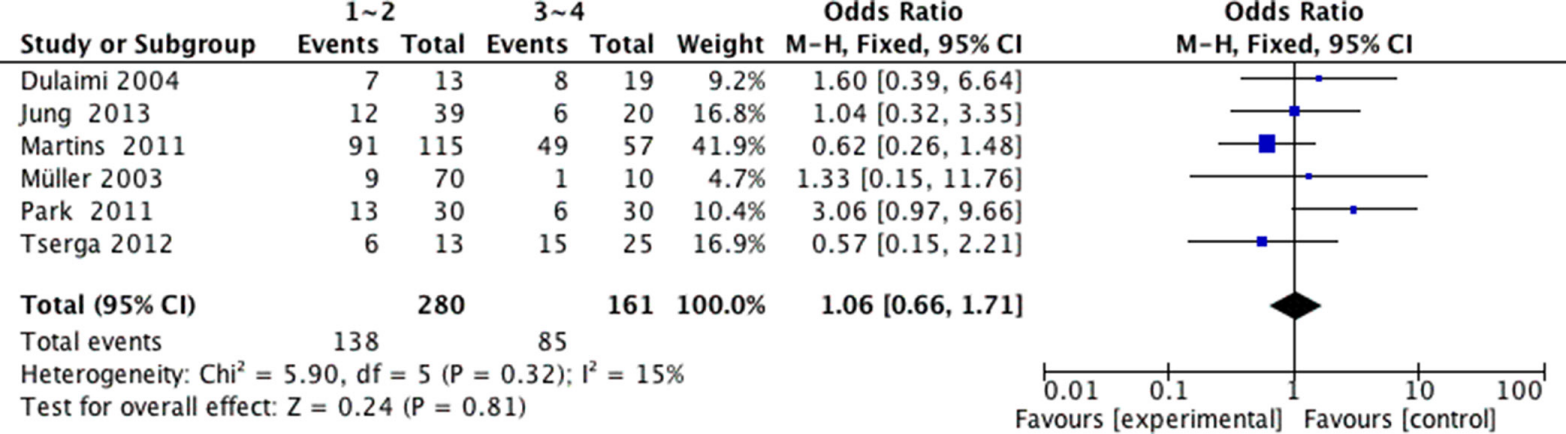
Forest plots of association between APC promoter methylation and cancer grade in breast cancer

**Figure 6:**
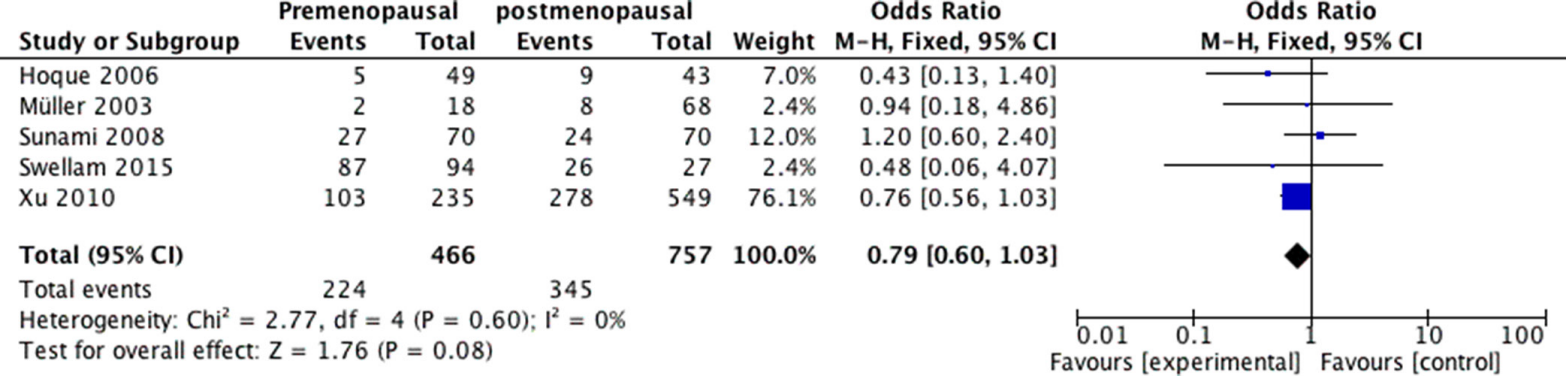
Forest plots of association between APC promoter methylation and menopausal status in breast cancer

**Figure 7:**
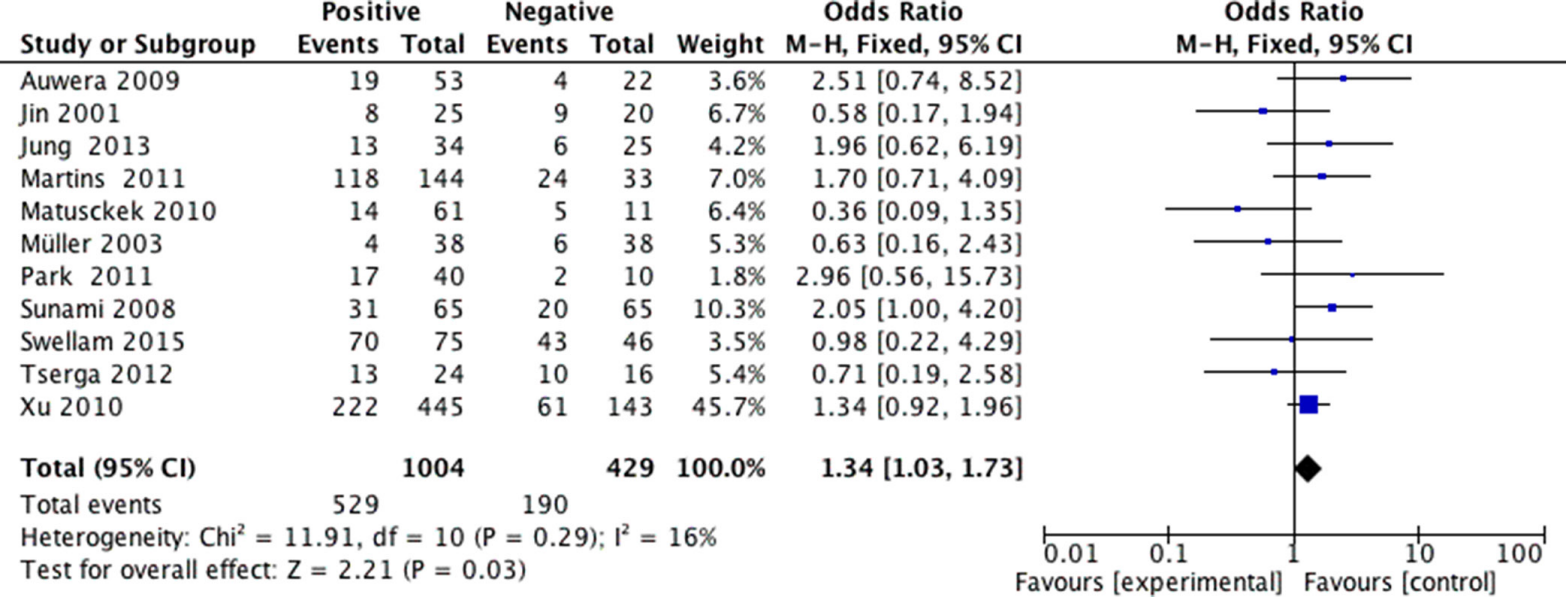
Forest plots of association between APC promoter methylation and ER status in breast cancer

**Figure 8:**
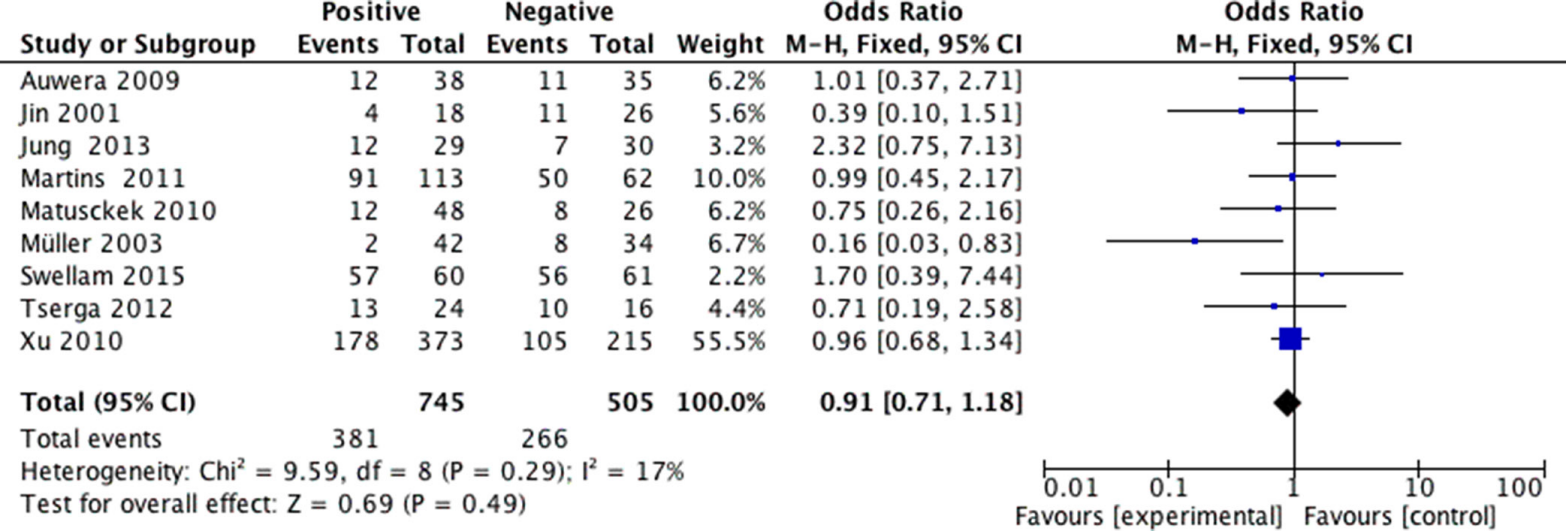
Forest plots of association between APC promoter methylation and PR status in breast cancer

**Figure 9:**
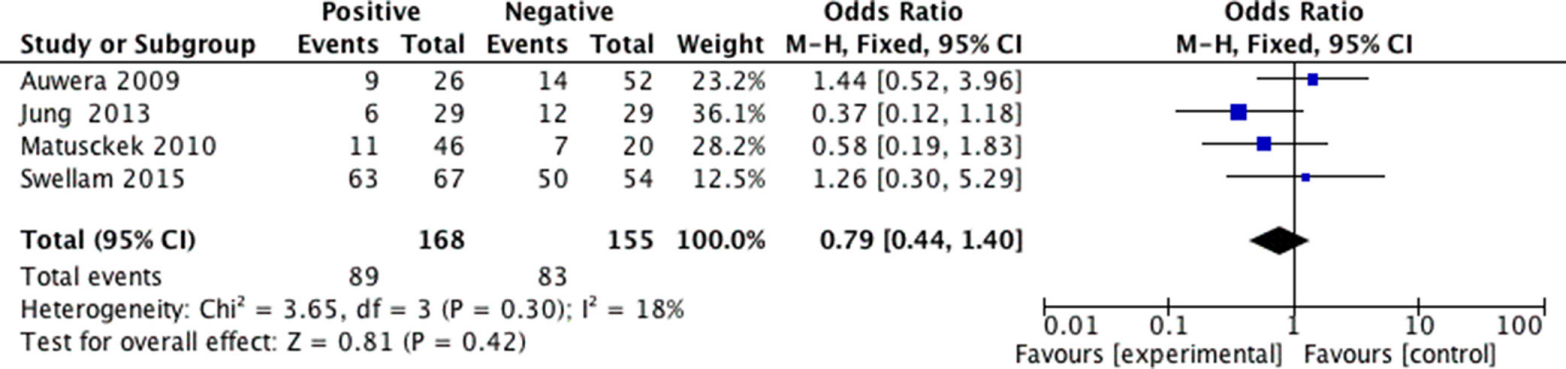
Forest plots of association between APC promoter methylation and HER-2 status in breast cancer

We analyzed 2293 samples from 15 studies to assess whether the abnormal APC methylation was associated with breast cancer clinicopathological characteristics, including cancer stage, cancer grade, lymph node metastasis, menopausal status, ER status, PR status and HER2 status. The result showed that the association between APC promoter methylation and cancer stage was significant (pooled OR = 0.47, 95% CI: 0.28–0.80, *P* = 0.006, Figure 3), and similar result existed in the association between APC promoter methylation and lymph node metastasis (pooled OR = 0.55, 95% CI: 0.36–0.84, *P* = 0.005, Figure 4), which both suggested that APC promoter methylation could inhibit the cancer growth and metastasis. However, we found that there was no significant association between APC promoter methylation and cancer grade (pooled OR = 1.06, 95% CI: 0.66–1.71, *P* = 0.81, Figure 5). There was no relationship between menopausal status and APC promoter methylation in breast cancer (pooled OR = 0.79, 95% CI: 0.60–1.03,*P* = 0.08, Figure 6). In analyses of ER, PR and HER-2, we demonstrated that there was an association between APC promoter methylation and ER (pooled OR = 1.34, 95% CI: 1.03–1.73, *P* = 0.03, Figure 7), inversely there was no relationship between APC methylation and breast cancer with PR^+^ (pooled OR = 0.91, 95% CI: 0.71–1.18, *P* = 0.49, Figure 8) and Her-2^+^ (pooled OR = 0.79, 95% CI: 0.44–1.40, *P* = 0.42, Figure 9), which was in consistent with the previous study by Sunami et al. [34].

### Association between APC promoter methylation and diagnosis of breast cancer

**Figure 10:**
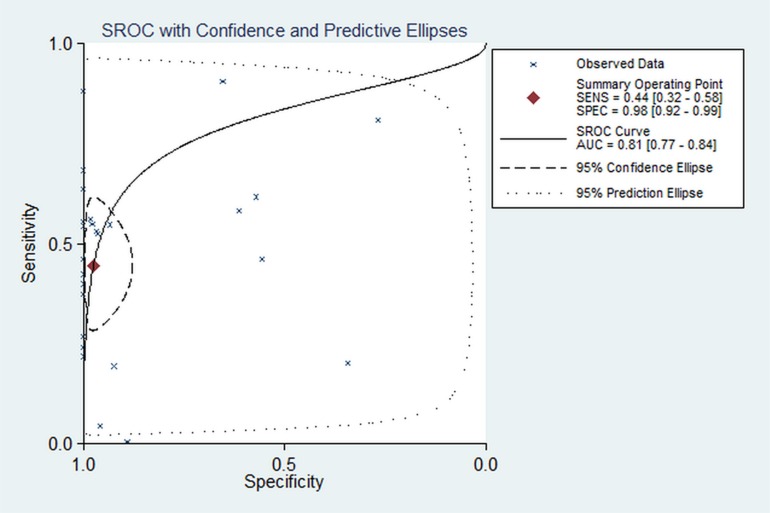
Meta-analysis with the S-ROC curve SENS: Sensitivity, SPEC: Specificity, AUC: Area under the curve.

The pooled sensitivity and specificity for all included studies were 0.444 (95% CI: 0.321–0.575, *P* < 0.0001) and 0.976 (95% CI: 0.916–0.993, *P* < 0.0001) based on mix-model. The AUC was 0.81 (95% CI: 0.77–0.84) (Figure 10), suggesting detecting APC methylation has a good diagnostic accuracy for breast cancers. In order to present more robust results on APC methylation as the detection marker, we conducted a subgroup analysis stratified by control type: the pooled OR of sensitivity and specificity for tissue sample group were 0.452 (95% CI: 0.302–0.611, *P* < 0.0001) and 0.982 (95% CI: 0.885–0.998, *P* < 0.0001); the pooled OR of sensitivity and specificity for blood sample group were 0.429 (95% CI: 0.232–0.651, *P* < 0.0001) and 0.962 (95% CI: 0.866–0.990, *P* < 0.0001). The AUC in all samples, tissue samples and blood samples analysis was 0.81, 0.79 and 0.85, which suggested that it is more appropriate to monitor the level of APC methylation in blood samples for the diagnosing breast cancers.

### Publication bias

**Figure 11:**
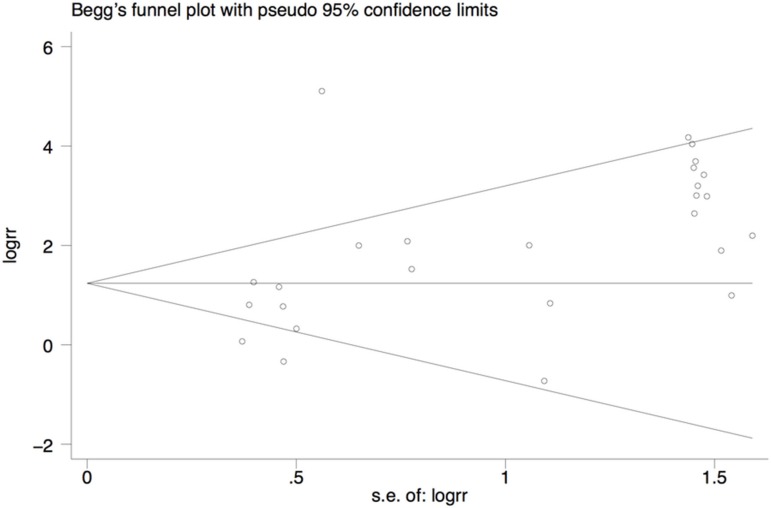
Begg's funnel plot for assessment of publication bias

We conducted potential publication bias by using Begg's rank correlation. The result suggested that there was no publication bias in breast cancer group versus control group (*P* = 0.052). Begg's funnel plot was shown in Figure 11.

## DISCUSSION

Breast cancer is a multiple steps, multiple phases, progressing procession of the tumourgenesis, and it is a type of gene disease involved in activation of oncogenes and inactivation of tumor suppressors [42]. As we know that APC is a tumor suppressor gene, which can be silenced or inactivated by methylation of CpG islands in gene promoter regions. Hence, we conducted a meta-analysis to investigate the association between breast cancer and APC methylation. Our data showed that the frequency of APC promoter methylation was demonstrated to be 5.92-fold high in breast cancer patients compared with non-breast cancer groups. The result was consistent with previous studies [12, 20–22, 24, 25, 43], which indicated that promoter methylation of APC could be implicated in the occurrence of breast tumors. Owing to the evident heterogeneity, we conducted stratification analyses based on methylation test method, control type and ethnicity. Finally we found that the value of *I*^2^ was reduced in all stratified analyses, which suggested that test method, control type and ethnicity could lead to the heterogeneity. To date, it has become apparent that biomarkers were associated with cancer clinicopathological characteristics [44–46]. In this meta-analysis, we revealed that the frequency of APC promoter methylation increased in advanced TNM stage and lymph node metastasis breast cancer but decreased in ER positive breast cacner. TNM stage and lymph node metastasis were two of the most important prognostic factors for breast cancer. As is known to all that the prognosis of patients can be greatly improved by endocrine therapy, and ER^+^ breast cancer patients have good response to hormonal therapy [47]. Therefore, we inferred that APC promoter methylation might be contribute to the corresponding biology and clinical outcome of breast cancer.

This meta-analysis showed that pooled sensitivity and specificity for all included studies were 0.444 and 0.976 respectively, suggesting that the APC promoter methylation is a valuable biomarker for diagnosis of breast cancer. The summary results of sensitivity and specificity were objective and easy to understand, but they were often affected by different threshold value. The SROC curve is a synthesized index that includes two indexes that reflect the accuracy of diagnostic test (sensitivity and specificity). Additionally, the SROC curve makes the location results much more accurate and scientific by considering the non-linear relation between the sensitivity and specific. Furthermore, the SROC curve can compare the diagnostic test through graph and AUC. The AUC close to 1.0 signifies that the test has almost perfect discrimination while an AUC close to 0.5 suggest poor discrimination in this study AUC in this study was 0.82. This was suggesting a good diagnostic accuracy of APC promoter methylation in breast cancer. However, APC promoter methylation may not be suitable for screening and diagnosing breast cancer alone due to the low sensitivity (OR = 0.444). The SROC curve in this meta-analysis showed that there was great difference in the sensitivity, suggesting that the results of sensitivity were unstable according to the way of the method and the experimental ability of investors. Therefore, continuously improving the experimental processes and methods may improve the sensitivity. Blood sample test is non-invasive and promising for clinical application because it is more acceptable to patients. In our analysis the AUC in blood sample test was the highest, indicating blood sample may be considered as the priority sample in the clinical application.

However, some limitations need to be discussed. Firstly, even though the heterogeneity decreased by subgroup analysis, it was still high. The difference of methylation detection primers may be one of the reasons. However we can't get enough information about the processes of detecting APC methylation from the included studies. Secondly, articles published in English were only included in this meta-analysis. We could have missed the articles published in other languages, because of anticipated difficulties in obtaining accurate medical translation. Thirdly, we did not retrieve articles related with APC methylation in different subtypes of breast cancer. Further studies should be needed to investigate the frequency of APC methylation in breast cancer types. Finally, there was low sensitivity for APC methylation to detect breast tumors. The reason is that a single tumor marker has limited power to diagnose breast cancers that implicate various genes and other factors. It is necessary to mention that the combination of several tumor makers can improve the sensitivity and specificity of diagnosis test for tumors. For instance, Brooks et al. [16] found that the diagnosis in combination with RASSF1A, GSTP1, RARb2, and APC methylation can significantly improve the detection of breast cancer.

In conclusion, this was the first meta-analysis about APC promoter methylation and breast cancer. The current evidence suggested that APC promoter methylation was associated with breast cancer risk and clinicopathological characteristics. In addition, APC promoter methylation was a valuable diagnostic biomarker with high specificity and qualified sensitivity. Well-designed prospective studies with larger sample sizes will help in further strengthening our observations.

## MATERIALS AND METHODS

### Search strategy and selection criteria

PubMed, EMBASE, and Cochrane Library were searched up to December 2015 using the key words “APC”, “methylation” and “breast cancer”. We also retrieved the reference lists of the articles identified in the searches for additional eligible studies. Studies included in the meta-analysis had to meet all the following criteria (1) the case–control or cohort studies assessing the association of APC methylation and breast cancer, (2) studies providing sufficient information to estimate odds ratio (OR) and 95% confidence interval (CI), (3) articles were published in English. The major reasons for exclusion of studies were (1) reviews, letter or case-only articles and (2) articles with insufficient data or duplicated data.

### Data extraction

Two investigators reviewed all of articles that fitted inclusion and exclusion criteria and independently extracted data from eligible studies. Disagreement was resolved by discussion and consensus. Data retrieved from the reports included author, year, country, ethnicity, control type, method for detecting methylation, clinicopathological characteristics, and number of methylated (M) and unmethylated (U) samples in cases and controls. We chose only one study with the largest sample size and the most detailed information when multiple reports were published from the same study population.

### Statistical analysis

Meta-analysis was preformed by Review Manager Software 5.2 (Cochrane Collaboration, Oxford, UK) and STATA software 11.0 (STATA Corp., College Station, TX, USA). OR with the 95% CI were used to examine differences in the frequency of APC methylation between breast cancer case and controls. The associations between APC methylation and breast cancer clinicopathological characteristics were also examined by the method. Data were extracted from the original studies and recalculated if necessary. *P*-value < 0.05 was considered to be statistically significant.

We used the sensitivity and specificity to estimate the diagnostic value of APC promoter methylation in breast cancer. However, the variation in the threshold definition of a positive result sometimes could produce an association between sensitivity and specificity values across studies. Thus we also constructed the summary receiver operator characteristic (SROC) curve that based on the sensitivity and specificity of each publication and calculated area under the SROC curve (AUC) to explore the diagnostic accuracy of APC methylation for breast cancer [48].

The heterogeneity among studies was checked with the Chi-square based on Q statistical test and *I*^2^. *P* ≤ 0.1 or *I*^2^ > 50% indicated significant heterogeneity among the studies and the pooled OR was estimated by the random-effects model (Mantel-Haenszel method). If the heterogeneity was in significant (*P* ≥ 0.1 or *I*^2^ < 50%), the fixed-effects model (inverse variance method) was employed. Publication bias was assessed by Begg's rank correlation.
